# Expert Group Consensus on early diagnosis and management of infantile-onset pompe disease in the Gulf Region

**DOI:** 10.1186/s13023-022-02545-w

**Published:** 2022-10-27

**Authors:** Zuhair Al-Hassnan, Nadia Al Hashmi, Nawal Makhseed, Tawfeg Ben Omran, Fatma Al Jasmi, Amal Al Teneiji

**Affiliations:** 1 Department of Medical Genetics, MBC-75 King Faisal Specialist Hospital & Research Center, Riyadh, Saudi Arabia; 2 Department of Child Health, National Genetic Center, Royal Hospital, Muscat, Sultanate of Oman; 3 Pediatric Department, Al-Farwaniya Hospital, and Maternity Hospital, Al-Jahra Hospital, Kuwait, Kuwait; 4 Division of Genetic and Genomic Medicine, Sidra Medicine, Doha, Qatar; 5 Department of Medical Genetics, Hamad Medical Corporation, Doha, Qatar; 6 Department of Genetics and Genomic Medicine, United Arab Emirates University, Abu Dhabi, United Arab Emirates; 7 Division of Metabolic Genetics, Department of Pediatrics, Tawam Hospital, Al Ain, United Arab Emirates; 8 Division of Metabolic Genetics, Department of Pediatrics, Sheikh Khalifa Medical City, Abu Dhabi, United Arab Emirates

**Keywords:** Cross-reactive immunologic material status, Enzyme replacement therapy, Gulf countries, Infantile-onset Pompe disease, Immunomodulation protocol

## Abstract

**Background:**

Infantile-onset Pompe disease (IOPD) is a rare and devastating, autosomal recessive lysosomal storage disorder that manifests immediately after birth. In severe IOPD cases, complete/almost-complete acid alpha-glucosidase enzyme deficiency is observed. Considering the rapid progression of the disease, timely diagnosis and treatment are important; even slight delays can remarkably alter the course of the disease. Enzyme replacement therapy (ERT) with recombinant human acid alpha-glucosidase is safe and beneficial for IOPD patients. However, there is heterogeneity in the patient response to ERT. The factors influencing treatment effectiveness include the patient’s age at the time of treatment initiation, pre-existing muscle damage, and cross-reactive immunologic material (CRIM) status at baseline. Immunomodulation along with ERT is the recently developed therapeutic approach that has been included in the therapeutic armamentarium of IOPD for optimizing clinical benefits, particularly in CRIM-negative IOPD patients. However, there is a dearth of published data on the early diagnosis and clinical position of the immunomodulation protocol along with ERT in the treatment of IOPD in the Gulf region.

**Methods and results:**

Expert panel meetings, involving six experts from the Kingdom of Saudi Arabia, Kuwait, Oman, Qatar, and the United Arab Emirates, were convened to develop consensus-based recommendations addressing current diagnostic and management challenges for patients with IOPD in the Gulf region. Furthermore, this consensus guideline may be implemented in clinical practice for the timely diagnosis and management of patients with IOPD.

**Conclusion:**

The expert consensus will help clinicians to make appropriate and timely decisions regarding immunomodulation initiation and ERT treatment in IOPD patients in the Gulf region.

**Take-home message**.

Immunomodulation in combination with ERT plays an effective role in the management of Pompe disease, particularly in patients with CRIM-negative status, when compared to ERT monotherapy.

## Background

Glycogen storage disease type II (OMIM 232,300), also known as Pompe disease, is a severe, autosomal recessive, and progressive genetic condition caused due to mutations in the acid alpha-glucosidase (*GAA*) gene, thereby leading to a reduction in the activity of the *GAA* enzyme in the lysosomes [1, 2]. Infantile-onset Pompe disease (IOPD) is a rare, devastating, and lysosomal storage disorder that manifests immediately after birth [2]. It is broadly classified into two categories: classic IOPD and nonclassic IOPD [3]. The clinical onset of the classic form of IOPD occurs usually before 12 months of age and is associated with clinical symptoms such as hypertrophic cardiomyopathy (HCM), hypotonia, and muscle weakness [1, 3]. The typical feature of the classic-onset form is the presence of HCM at birth, whereas this symptom is presented several months later in the case of nonclassic IOPD [3].

The global incidence of IOPD is commonly reported to be 1 in 150,000 births [4]. Clinical studies elucidate that 28% of Pompe disease cases are IOPD cases [5, 6]. Of these, 85% of cases comprise the classic infantile-onset form and 25% of these patients have a cross-reactive immunologic material (CRIM)-negative status [5, 6]. Ethnicity appears to exhibit varying effects on the incidence of Pompe disease [7]. The estimated frequencies of IOPD are 1 in 138,000 in the Caucasian population, 1 in 50,000 in the Chinese population, and 1 in 31,000 among those of African ancestry [7]. Epidemiologic studies in the Middle East region indicate a rising IOPD burden in the Gulf region. In particular, the prevalence of IOPD in the United Arab Emirates (UAE) has been reported as 2.66 per 100,000 [8]. There is a scarcity of population-based data on the incidence of IOPD in the Kingdom of Saudi Arabia (KSA) [9]. It is noteworthy that the possible reason for the high incidence of the disease may be the high degree of consanguinity in the region [9]. Therefore, there is a need for early and appropriate diagnostic and treatment strategies for the management of IOPD.

Underdiagnosis, poor recognition, and diagnostic delays are significant barriers to the early and accurate diagnosis of IOPD [10]. Lagler et al. investigated the diagnostic delays in Pompe disease based on the opinions of patients, parents, and physicians. The key findings of this study group are as follows: (1) metabolic specialists or pediatricians play a pivotal role in the accurate diagnosis of the disease rather than other physicians who hold expertise in other specialties, and (2) direct referral of patients to metabolic expert centers reduces diagnostic delays considerably rather than when several physicians having expertise in different areas are consulted for diagnosis. Therefore, direct referral to expert centers is a novel diagnostic strategy that can assist in early disease diagnosis [10].

Enzyme replacement therapy (ERT) with alglucosidase alfa is the approved therapeutic option for managing IOPD. Prompt treatment initiation through early diagnosis could certainly reduce the morbidity and mortality associated with the disease. The varied clinical effects associated with ERT are due to several factors, such as the age when treatment was initiated, dosage, severity of the disease, and prescribed course of ERT treatment [3]. Study findings of a 52-week, open-label study depicted a mortality rate of 33.3% (6/18) in infants with Pompe disease who received ERT [11]. Additionally, study findings of Chakrapani et al. reported a mortality rate of 35% (7/20) in ERT-received infants with Pompe disease [12]. A recent retrospective study conducted on 18 Saudi patients concluded that, despite effective ERT treatment, the mortality rate was 83.3% (15/18) [1]. The determination of the CRIM status of infants with Pompe disease is considered extremely important because it plays a pivotal role in deciding the ERT dose, thereby potentially improving patient outcomes [1, 5]. The significant drawback associated with ERT is the development of immunoglobulin G (IgG) antibody titers against ERT, which reduces treatment efficacy and safety outcomes [13]. On the one hand, in the case of CRIM-negative patients who lack or do not produce endogenous *GAA* protein at all, the infused enzyme is identified as a foreign protein by the patient’s immune system and the patient’s body produces high titers of neutralizing antibodies, which deteriorate the clinical benefit of ERT in the later course of treatment, although an initial improvement is seen [2]. On the other hand, in the case of CRIM-positive patients who have specific levels of endogenous *GAA*, the infused recombinant human acid alpha-glucosidase (rhGAA) is not identified as a foreign protein and the neutralizing antibody titer production remains low, leading to effective ERT treatment [2]. To overcome this problem of neutralizing antibody titer production, a novel strategy has been developed in the therapeutic armamentarium of Pompe disease, which is immunomodulation therapy or immune tolerance induction (ITI) [14]. This protocol has proved to induce immune tolerance to ERT, thereby improving overall survival, particularly in ventilator-dependent IOPD patients with CRIM-negative status who developed IgG antibodies when managed with ERT monotherapy [13]. Even in patients with CRIM-positive status, coadministration of immunomodulation therapy along with ERT decreased the development of IgG antibodies compared to cases managed with ERT monotherapy [13].

In the Gulf settings, consensus-based guidelines from the Middle East and North Africa region had provided practical recommendations regarding the initiation of ERT and diagnostic and treatment algorithms for late-onset Pompe patients [15]. These guidelines recommended an alglucosidase alfa dosage of 20 mg/kg body weight administered every other week (EOW) as an intravenous infusion for the management of late-onset Pompe disease (LOPD) [15]. It is noteworthy that there is a dearth of such recommended guidelines for the diagnosis and treatment of IOPD in the Gulf region. Therefore, it is necessary to publish consensus-based guidelines to ensure an up-to-date frame of reference for healthcare professionals and patients. Furthermore, considering the CRIM status, several concerns on the management of IOPD with ERT and immunomodulation protocol remain unanswered. A thorough investigation of the role of the immunomodulation protocol in the management strategies of IOPD is crucial in the Gulf settings as there is a lack of qualitative evidence on the management of IOPD with immunomodulation protocol and ERT in this Gulf region.

With this background, an effort was made to understand the unmet clinical challenges of diagnostic and treatment approaches for IOPD patients, especially from the Gulf perspective. Based on their clinical knowledge and after careful evaluation of available evidence, the panelists presented their expert opinions and views regarding the diagnosis and management of IOPD patients. Therefore, the present consensus document aims at giving the complete picture of the Pompe disease situation to clinicians across the Gulf region to better diagnose and manage IOPD patients, especially with the appropriate use of novel treatment strategies, such as immunomodulation protocol.

## Methods

**Fig. 1:**
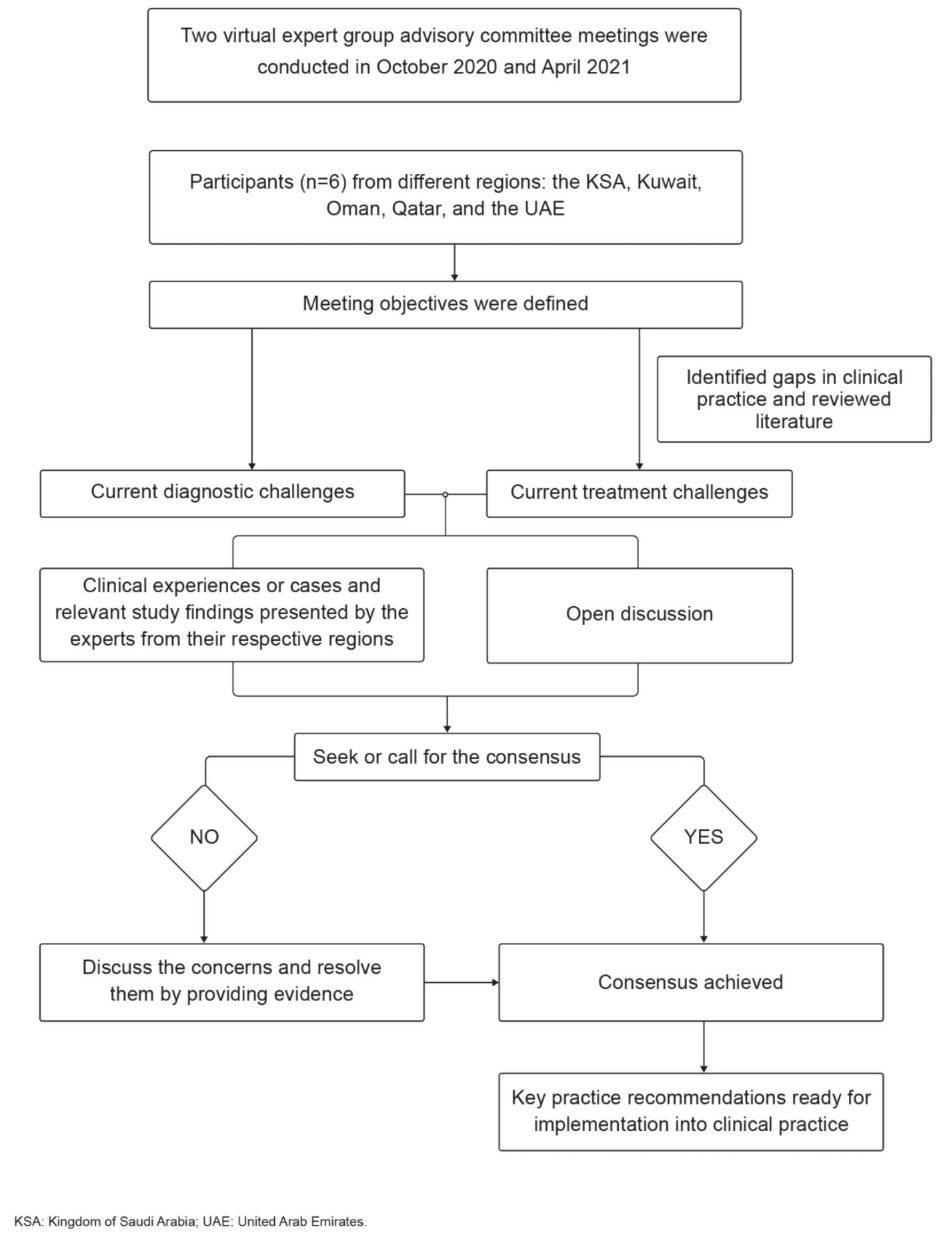
Flowchart representation of consensus development

The expert group advisory committee meeting, encompassing six experts from the KSA, Kuwait, Oman, Qatar, and the UAE, was convened in October 2020 and April 2021 to generate a list of recommendations on the following Gulf concerns: current diagnostic and management challenges of IOPD. The meeting was conducted based on the request from advisors to develop recommendations to fill the current gaps in IOPD management in the Gulf region. The consensus was developed based on expert insights on the early diagnostic challenges and management dilemmas related to the initiation and dosage of ERT in CRIM-negative and CRIM-positive patients and the introduction of immunomodulation protocol in IOPD patients. The structural representation of the consensus development is presented in Fig. 1. The experts reached a consensus regarding the diagnostic and management approaches of IOPD.

## Results

The meeting recommendations were based on the consensus reached by the participants, which was supported by their clinical experience and research findings.

### Evidence for current diagnostic approaches of IOPD

Early diagnosis is a significant factor in the effective management of IOPD. Various ancillary tests and diagnostic methods are presented in Table 1 [2, 9, 16]. Chest X-ray; electrocardiogram; echocardiogram; electromyography; muscle magnetic resonance imaging; and nonspecific laboratory biomarkers including blood tests, such as creatine phosphokinase, lactate dehydrogenase (LDH), alanine transaminase (ALT), and aspartate transaminase (AST), and urine tests, such as glucose tetrasaccharide (HEX4), should be done in clinically suspected infants with Pompe disease [2, 16]. *GAA* activity in dried blood spots (DBS) is a good practical screening test for Pompe disease, whereas the diagnostic workup includes *GAA* activity in lymphocytes or leukocytes and performing mutation testing [2, 16].

**Table 1:** Laboratory tests and diagnostic methods to confirm IOPD

Diagnostic approaches	Confirmatory findings
CPK	Elevated (not more than 1500–2000 IU/L)
Blood tests, such as ALT, AST, LDH, and urine tests, such as HEX4	Elevated
Chest X-ray	Reduction in lung volume, cardiomegaly, and/or areas of atelectasis
Electrocardiogram	Short PR interval, inverted T-wave, wide/broad QRS complex
Echocardiogram	Hypertrophic cardiomyopathy with or without ventricular outflow tract obstruction; the late stages of the disease are often characterized by dilated cardiomyopathy
MRI (not routinely indicated)	Greater axial muscle involvement; complete involvement of lumbar paravertebral muscles and psoas; T1-weighted images are sufficient for an adequate assessment
Muscle biopsy (histopathology)	Severe glycogen storage and vacuolar myopathy, positive acid phosphatase, positive PAS staining, and reduced myofibrils

ALT: Alanine transaminase; AST: Aspartate transaminase; CPK: Creatine phosphokinase; HEX4: Glucose tetrasaccharide; IU/L: International units per liter; IOPD: Infantile-onset Pompe disease; LDH: Lactate dehydrogenase; MRI: Magnetic resonance imaging; PAS: Periodic acid Schiff

### Current clinical challenges in the diagnosis of IOPD

Initial diagnostic delays are significant barriers; specifically, enzyme assay tests can extend up to 6 weeks and can delay the diagnosis of a disease [2]. In particular, based on the clinical experience of experts in the UAE region, the diagnostic delay is a major reason for death in IOPD patients. In addition, the diagnosis itself is clinically challenging due to the heterogeneous presentation of nonspecific symptoms, which may overlap with other myopathies or neuromuscular disorders [17]. The symptoms of IOPD that mimic other diseases have been presented in Table 2 [16, 18]. These nonspecific clinical features, including HCM, respiratory insufficiency, hypotonia, and myopathy, hinder initial diagnosis and thereby cause diagnostic delays [19]. Therefore, differential diagnoses play a crucial role in the accurate diagnosis of IOPD. Besides diagnostic delays, variants of unknown significance are also a significant hurdle for early diagnosis as these need to be further analyzed for the initiation of treatment [15]. Physicians who have expertise in metabolic and neuromuscular diseases, with support from a skilled multidisciplinary team, can overcome the diagnostic challenges to a great extent.

**Table 2:** Differential diagnoses of IOPD

Shared symptoms between IOPD and other disorders	
Disorder	Shared symptoms with IOPD
Spinal muscular atrophy | (acute Werdnig–Hoffman disease)	Hypotonia
	Progressive proximal muscle weakness
	Absence of reflexes
	Difficulties in feeding
	Elevated levels of CK
Congenital muscular dystrophy	Severe hypotonia
	Severe weakness in muscles
Hypothyroidism	Hypotonia
	Enlarged tongue (macroglossia)
Endocardial fibroelastosis	Dyspnea (breathlessness)
	Difficulties in feeding
	Enlarged heart (cardiomegaly)
	Heart failure
Danon disease	Hypertrophic cardiomyopathy
	Skeletal muscle myopathy
	Vacuolar glycogen storage
	Elevated levels of CK
Carnitine deficiency	Cardiomyopathy
	Weakness in muscle
Glycogen storage diseases III and IV	Enlarged heart (cardiomegaly)
	Weakness in muscle
	Elevated levels of CK
	Enlarged liver (hepatomegaly)
	Hypotonia
Idiopathic hypertrophic cardiomyopathy	Biventricular hypertrophy
Myocarditis	Enlarged heart (cardiomegaly)
Mitochondrial/respiratory chain disorders	Enlarged liver (hepatomegaly)
	Cardiomyopathy
	Myopathy
	Elevated levels of CK
Peroxisomal disorders	Hypotonia
	Enlarged liver (hepatomegaly)

CK: Creatine kinase; IOPD: Infantile-onset Pompe disease

### Consensus statement on diagnostic approaches to IOPD in the Gulf region

- In the presence of a family history of confirmed diagnosis and phenotype that is consistent with IOPD with hypotonia, HCM, and hyperCKemia, an initiation of treatment is recommended even before confirmatory testing is available.
- In the absence of a family history of IOPD, the expert group recommendations are as follows:

- Enzyme activity on DBS plus *GAA* screening for known founder mutations in the family/tribe.
- If the results of DBS testing and mutation testing are positive, then initiation of treatment is recommended.
- In the absence of known founder mutations, full *GAA* testing is recommended.
- Based on the mutation results, CRIM status can be predicted. If the CRIM status associated with the mutation is not known, then the determination of CRIM status is recommended.

- Late diagnosis is a significant barrier associated with Pompe disease. The confirmation of Pompe disease is the biggest challenge, even with a greater clinical disease suspicion. In the majority of cases, a clinical decision is reached based only on genetic testing (targeted mutation analysis) as enzyme assay test reports are received usually after treatment initiation.
- As per the local guideline-based approach, family history plays an important role in diagnosing Pompe disease. In a newborn with positive family history, if the clinical phenotype is highly suggestive of Pompe disease with hypotonia, HCM, and hyperCKemia and the history suggests a confirmed Pompe disease case, treatment initiation is strongly recommended even before confirmatory testing is available. However, it should be done only under the supervision of an expert in the management of IOPD.
- In the presence of a previously affected child with Pompe disease, prenatal testing is crucial for early diagnosis, which may enable early treatment. Pompe disease is a potentially fatal illness; hence, in some centers, termination of pregnancy is offered. The treating physician should follow the country’s law and hospital regulations in this regard.

### Newborn screening can be a screening tool for the diagnosis of IOPD

Newborn screening (NBS) can be an excellent approach for early diagnosis and timely treatment initiation. Early diagnosis and implementation of timely treatment can change the natural history of the disease and, to a great extent, reduce mortality [20]. NBS is now becoming a platform for the diagnosis of IOPD in several countries. After the NBS program was devised in Taiwan, from 2016 to 2019, 100% of newborns were screened and 14 infants were detected to be at a high risk of Pompe disease, whereas classic IOPD was diagnosed in 4 infants. Overall, the Taiwan Pompe disease screening and diagnostic programs have led to prompt and successful diagnosis and management of IOPD patients [20]. The NBS program conducted in Illinois among 684,290 infants during 2015–2019 identified 395 infants (0.06%) with positive screening for Pompe disease. Upon further diagnostic assessment, a total of 29 patients were confirmed with Pompe disease, of which 3 patients were with IOPD and 26 patients with LOPD [21]. In the Middle East region, the incidence of inherited metabolic diseases is common due to a high consanguinity rate of 25–70% and a greater extent of first-cousin marriages [22]. Therefore, there is a need for the initiation of preventive steps by developing nationwide NBS strategies in this region based on the experiences of regional and international level screening programs.

### Consensus statement on the NBS approach

- There are limited pilot studies conducted in the Gulf region. Pilot studies are highly recommended as they may help in analyzing data and expecting the outcome and may be indicative of the cost-effectiveness of performing NBS in these cases. Adequate laboratory infrastructure is very important for NBS, which is lacking in the Gulf region, to perform screening for Pompe disease.
- In addition, there are insufficient data concerning the natural history of the disease, percentage of LOPD, long-term outcomes of CRIM-negative cases, and the outcomes of intervention with immunomodulation in the Gulf region as they are time-consuming studies. Furthermore, there are no clear guidelines on when to start treatment for LOPD cases detected by NBS.
- Currently, the NBS approach for Pompe disease is not a priority. Thus, it would be very difficult to make a recommendation for universal screening for Pompe disease. However, the expert group recommends the periodic evaluation of Pompe disease in the NBS programs in the regions, if conditions permit.
- It is very important to identify high-risk groups, including patients with early-onset infantile HCM and positive family history. These high-risk groups can help in identifying high-risk patients by focusing on IOPD and thereby helping in the early initiation of treatment.
- As a part of preventive measures, not only prenatal diagnosis but also premarital and preimplantation genetic diagnosis can be offered in extending carrier screening for at-risk family members. Performing such focused preconception and prenatal screening of these families will significantly help in the prevention of Pompe disease in the subsequent generations.
- Obstetricians and fetomaternal specialists should play a pivotal role in suspecting Pompe disease by the identification of cardiomegaly through prenatal screening during pregnancy.

### Evidence on current management approaches of IOPD

Pompe disease is a multisystemic condition; thus, a multidisciplinary team, headed by a metabolic specialist with expertise in the management of IOPD, is quite essential for its effective management [16]. Although an established ERT protocol is available for the management of IOPD, a supportive or nonspecific treatment measure should also be part of the management based on cardiac, pulmonary, neurologic, musculoskeletal, and gastrointestinal symptoms. The specific treatment approved for the management of IOPD is ERT with rhGAA at the standard dose (20 mg/kg/EOW) [1, 2, 17]. However, there is no strong evidence on which dosing regimen, that is, 20 mg/kg or 40 mg/kg, is effective for the management of IOPD [23] because there are studies supporting the optimal efficacy with the 40 mg/kg dosing schedule of ERT [3, 24, 25]. Even a recent European Pompe Consortium’s observational cohort study emphasized that the survival rate significantly improved in IOPD patients treated at a high dose (40 mg/kg/ week) than that in patients who were managed with a standard dose (20 mg/kg/EOW) [26]. Furthermore, poor survival rates or patient outcomes, such as a decline in motor function, were associated with ERT standard dose [22]. However, the CRIM status of the patient significantly affects treatment outcomes and will decide the ERT dosing regimen [2, 27]. Overall, evidence from real-world experiences depicted that early and high doses of alglucosidase alfa improve outcomes in IOPD patients [3].

It is known that high neutralizing antibody titers develop in some CRIM-positive and all CRIM-negative patients under ERT [27, 28]. The clinical benefit of ventilator-free survival in high-titer CRIM-positive and high-titer CRIM-negative patients is very poor and is similar to that of untreated patients. Furthermore, based on the clinical experience of experts in the Oman region, in some cases, severe phenotype CRIM-negative status with high antibody titers is the reason for mortality despite the early diagnosis. In some cases, an unknown CRIM status with suboptimal rhGAA dosing can also be the reason for death. Therefore, to prevent the development of high IgG antibody titers in some CRIM-positive patients and all CRIM-negative patients, a prophylactic immunomodulation therapy or ITI was proposed [14]. Rapid determination of the CRIM status is essential because ITI must begin before the initiation of ERT, but the CRIM test should not postpone the initiation of ERT. In conclusion, treatment with ITI plus ERT has to be started in patients with IOPD with unknown CRIM status [28].

Based on the existing literature, the recommended ITI protocol lasts for approximately 5 weeks and includes one course of intravenous rituximab 375 mg/m^2^ (if body surface area is < 0.5 m^2^, then 12.5 mg/kg) weekly four times; subcutaneously methotrexate 0.4 mg/kg daily for the first 3 days of a week along with first three ERT infusions, thus a total of nine doses, with or without intravenous immunoglobulin [14, 25]. This intravenous immunoglobulin (400–500 mg/kg) is administered every month for 5–6 months. The addition of bortezomib in patients with established high antibody titers has shown beneficial effects in reducing antibody titers [29]. The study findings of Banugaria et al. and Kazi et al. demonstrated the clinical benefits of an ITI regimen when it was started along with ERT [14, 30]. Even the results of a retrospective study conducted among 34 IOPD (9 CRIM-positive and 25 CRIM-negative) patients demonstrated that a short course of prophylactic ITI protocol along with ERT was effective when compared to ERT alone [13]. Thus, prophylactic ITI protocols before ERT initiation or simultaneous initiation of ERT and ITI protocols is considered a standard of care for IOPD patients.

### Current clinical challenges in the treatment of IOPD

The first and foremost challenge in the treatment of IOPD is the response of patients with IOPD to ERT as it is very heterogeneous. To date, no international studies have been conducted to evaluate the long-term clinical outcomes of ERT in a large cohort of IOPD patients [3, 31]. Late initiation of ERT is a significant barrier that is associated with poor patient outcomes [25]. The development of anti-rhGAA IgG antibodies is another challenge; however, ITI protocol can be a solution as it may improve the poor outcomes of ERT [32]. The other unmet needs are the treatment of neurocognitive problems in patients with IOPD with ERT because rhGAA does not pass through the blood–brain barrier [31]. Overall, early initiation of ERT concurrently with ITI protocol would to some extent resolve treatment-related challenges of IOPD.

### Consensus statement on treatment approaches of IOPD in the Gulf region

- Patients with IOPD should be managed as early as possible with immunomodulation plus a low-dose ERT (20 mg/kg/EOW). However, a dose increment to 40 mg/kg (EOW [off-label]) plus immunomodulation is suggested if poor outcomes are noted.
- However, emerging data based on international as well as regional experiences demonstrate that a dose increment to 40 mg/kg/week plus immunomodulation may have a good outcome, especially in Pompe patients with CRIM-negative status. However, the expert group agrees that a dose increment to 40 mg/kg/weekly (off-label use) should be individualized and should only be done in a center with experience in treating Pompe disease (should be given with caution) [33].
- In CRIM-positive Pompe patients with a mutation of mild effect and with less severe phenotype presentation, a lower dose of ERT (20 mg/kg/EOW) is suggested. Higher doses (20 mg/kg/weekly [off-label]) may be considered in some patients based on their clinical assessment and response.

### Key expert opinion for practice

- IOPD is a debilitating, progressive, and mostly fatal neuromuscular disorder with a broad spectrum of clinical phenotypes.
- A clinical confirmation is reached based only on genetic testing (targeted mutation analysis).
- In a newborn with positive family history, treatment initiation is strongly recommended even before confirmatory testing is available if the clinical phenotype is highly suggestive of Pompe disease with hypotonia, HCM, and hyperCKemia, and the history suggests a confirmed Pompe disease case.
- The main aim of ERT is to prevent, reverse, stabilize, or slow the progression of the disease depending on the disease stage and severity at the time of diagnosis and treatment initiation.
- Early diagnosis and treatment predict the outcomes of IOPD.
- Immunomodulation protocol should be initiated in all patients with IOPD as it significantly improves outcomes, mortality, and morbidity. Specifically, immunomodulation protocol should be initiated along with ERT in patients with a CRIM-negative status, severe phenotype, and early disease onset. Overall, immunomodulation in combination with ERT plays an effective role in the management of Pompe disease with CRIM-negative status when compared to ERT monotherapy.
- An individualized, stepwise approach is recommended for IOPD management, and the dose of ERT depends on the severity of clinical phenotype and CRIM status. Initially, treatment should be started with 20 mg/kg EOW in combination with the ITI regimen, irrespective of CRIM status. However, there is a need for a higher ERT dose (40 mg/kg/weekly) in combination with ITI if significant improvement in clinical outcomes was not reported with a lower ERT dose (20 mg/kg/EOW). Therefore, immunomodulation initiated before a higher dose of ERT (40 mg/kg/weekly) may improve the outcomes of IOPD patients with greater severity and should be done under the supervision of an expert with a multidisciplinary team. If the CRIM status is unknown, then an aggressive approach has to be initiated as if it is CRIM-negative and the treatment strategy could be modified once the CRIM status is confirmed.

### Important considerations of immunotherapy

- Mutation status should be known to predict the CRIM status. Assessment of CRIM status should not delay the initiation of treatment of confirmed or highly suspected IOPD cases.
- Anti-rhGAA antibodies should be quantified at baseline and regularly by the primary physician throughout treatment.
- Repetition course of immunotherapy is determined by increasing anti-rhGAA antibodies to > 6400 on two or more occasions, and a cluster of differentiation 19% (CD 19%) recovery at more than 5 months after the last immunotherapy.
- Laboratory investigations in conjunction with ITI protocol should be performed as per published data [16].

## Discussion

**Fig. 2:**
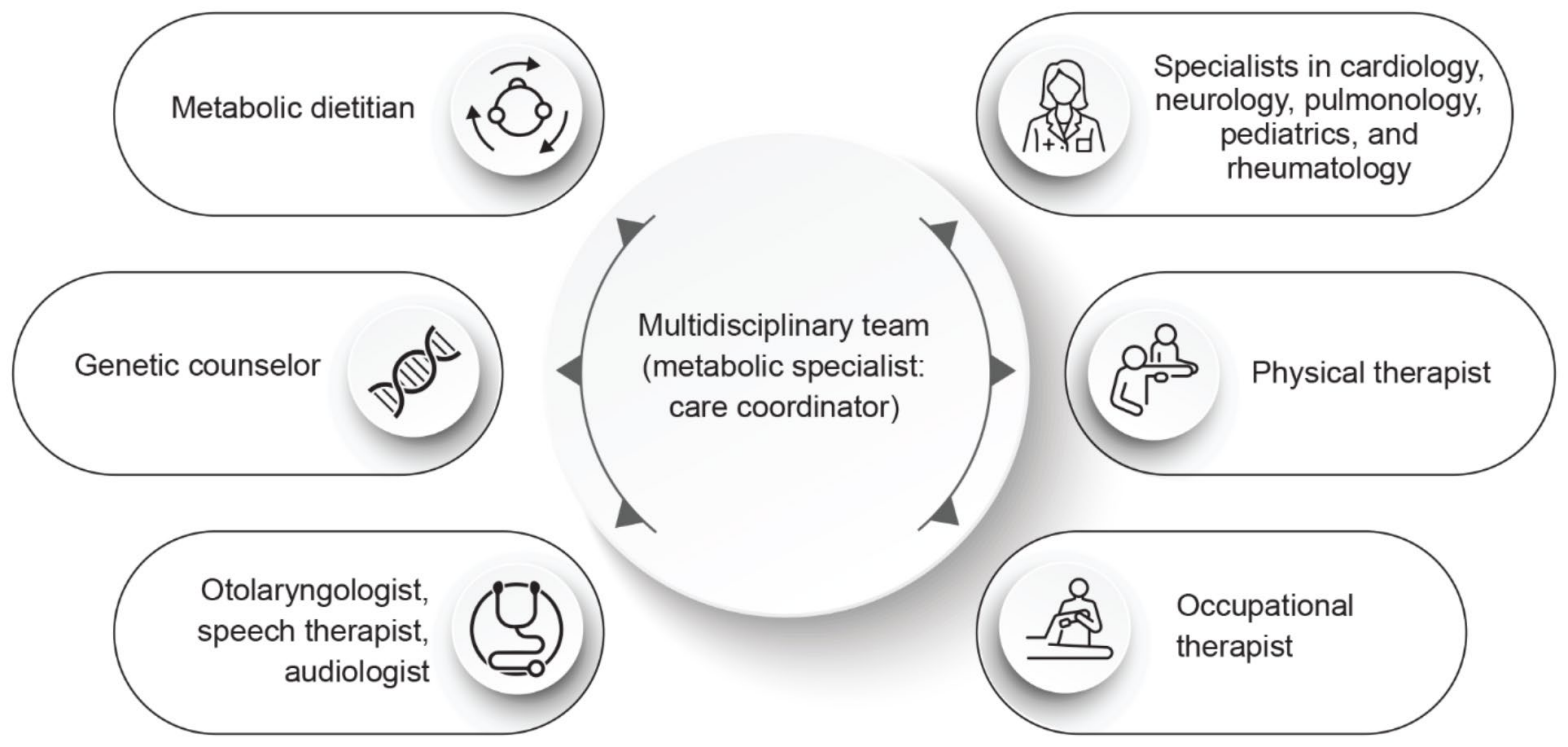
Multidisciplinary team for the management of patients with IOPD.

IOPD is a multisystemic condition that develops multiple clinical manifestations, including symptoms related to cardiovascular, gastrointestinal, neurologic, and pulmonary systems, in the first year of life [16]. Therefore, for the proper management of IOPD, a multidisciplinary team with sound knowledge of the natural history of the disease and associated challenges, including the emotional and psychological effects of the disease on families of IOPD patients, is necessary (Fig. 2) [16].

The treatment strategy available for the management of IOPD is ERT with alglucosidase alfa, which has changed the disease course in IOPD patients [34]. The recent introduction of the ITI protocol into the therapeutic armamentarium of IOPD, in combination with ERT, has substantially changed the disease course and has shown improved survival outcomes when compared to that of ERT alone in patients with IOPD [13].

To our knowledge, this document is the first to give region-specific consensus-based guidelines for the Gulf region on IOPD diagnosis and management, considering the diagnostic and treatment challenges in the region. This region-specific consensus manuscript aids physicians by facilitating early diagnosis, effective treatment, and prompt monitoring, which may provide optimal clinical benefits. It is noteworthy that underdiagnosis, delay in diagnosis, and delay in treatment are the consequences of a lack of awareness about the disease in the region. Therefore, increasing awareness among physicians may ensure prompt diagnosis and early treatment initiation in diagnosed IOPD patients.

Besides increasing awareness in healthcare professionals, educating patient families about the disease is needed as they are reluctant to consult a physician in the early stages of the disease, which may lead to a delay in diagnosis and poor treatment outcomes. It is important to increase awareness of the importance of family screening in high-risk families. Overall, parents of patients need to seek medical advice of the patient during the early and mild course of the disease, and lifelong treatment without any refusal is needed for effective outcomes. The experts hope that the dissemination of these consensus reports will aid clinicians in attaining a prompt diagnosis and facilitating effective management and proper monitoring of patients with IOPD to minimize the disease burden.

## Conclusion

The management of IOPD is challenging as ERT has not shown satisfactory results. Based on existing literature, immunomodulation therapy may be effective in treating IOPD patients. In the Gulf region, there is a dearth of qualitative evidence on the position of immunomodulation agents in the treatment of IOPD. The current expert-based consensus, along with the clinical experience shared by experts, would establish comprehensive and practical approaches for the positioning of ERT and the immunomodulation protocol in the management algorithm of IOPD in clinical settings. NBS offers an early diagnosis and presymptomatic ERT initiation. It prevents cardiac and respiratory complications and helps in achieving normal growth and development in patients with IOPD.

## Data Availability

Not applicable.

## Abbreviations

ALT: Alanine transaminase.
AST: Aspartate transaminase.
CPK: Creatine phosphokinase.
CRIM: Cross-reactive immunologic material.
EOW: Every other week.
ERT: Enzyme replacement therapy.
*GAA*: Acid alpha-glucosidase.
HEX4: Glucose tetrasaccharide.
HCM: Hypertrophic cardiomyopathy.
IU/L: International units per liter.
IgG: Immunoglobulin G.
IOPD: Infantile-onset Pompe disease.
ITI: Immune tolerance induction.
KSA: Kingdom of Saudi Arabia.
LDH: Lactate dehydrogenase.
LOPD: Late-onset Pompe disease.
MRI: Magnetic resonance imaging.
NBS: Newborn screening.
rhGAA: Recombinant human acid alpha-glucosidase.
UAE: United Arab Emirates.
