# Correction: Expert Group Consensus on early diagnosis and management of infantile-onset pompe disease in the Gulf Region

**DOI:** 10.1186/s13023-023-02967-0

**Published:** 2023-11-23

**Authors:** Zuhair Al-Hassnan, Nadia Al Hashmi, Nawal Makhseed, Tawfeg Ben Omran, Fatma Al Jasmi, Amal Al Teneiji

**Affiliations:** 1 Department of Medical Genetics, MBC-75 King Faisal Specialist Hospital & Research Center, Riyadh, Saudi Arabia; 2 Department of Child Health, National Genetic Center, Royal Hospital, Muscat, Sultanate of Oman; 3 Pediatric Department, Al-Farwaniya Hospital, and Maternity Hospital, Al-Jahra Hospital, Kuwait, Kuwait; 4 Division of Genetic and Genomic Medicine, Sidra Medicine, Doha, Qatar; 5 Department of Medical Genetics, Hamad Medical Corporation, Doha, Qatar; 6 Department of Genetics and Genomic Medicine, United Arab Emirates University, Abu Dhabi, United Arab Emirates; 7 Division of Metabolic Genetics, Department of Pediatrics, Tawam Hospital, Al Ain, United Arab Emirates; 8 Division of Metabolic Genetics, Department of Pediatrics, Sheikh Khalifa Medical City, Abu Dhabi, United Arab Emirates

**Correction: Orphanet Journal of Rare Diseases (2022) 17:388** 10.1186/s13023-022-02545-w

Following publication of the original article [1], we have been notified that the corresponding author name was published incorrectly. It was Zuhair Al Hassnan and it should be as follows:

Zuhair Al-Hassnan
